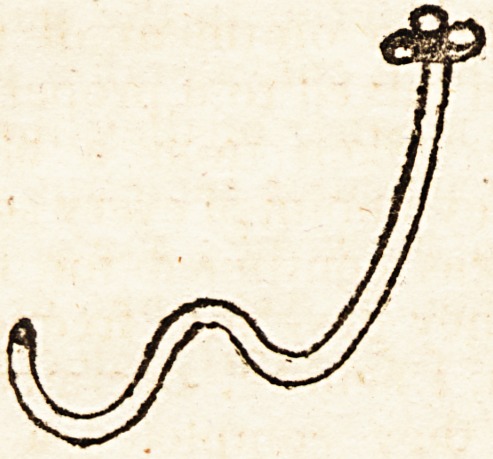# Singular Shape of a Catheter, When Withdrawn

**Published:** 1808-02

**Authors:** 


### *To the Editors of the Medical and Phyſical Journal.*

GENTLEMEN,

BEING lately called in a case of suppression of urine, where the elastic Catheter was introduced, I was astonished to observe the extraordinary shape of the instrument when withdrawn. As the operation was performed by a surgeon long established in practice, and who has for some time held a situation of high responsibility, I can impute the very singular shape of the catheter to no other cause than to a mal-conformation of the urethra: a further proof of this is, that several bougies that were introduced, exhibited when withdrawn the same tortuosity of shape. Had this happened in the hands of a young practitioner, suspicions might have arisen in our minds of the correctness of the introduction; but the *rank* alone of the gentleman is fully sufficient to stifle every doubt of his ability to perform such a common operation; leaving us fully warranted to conclude that a mal-conformation actually existed in the urethra.

The shape of the catheter when withdrawn from the urethra exhibited the following appearance.

From the singularity of the cure, as well as from *particular considerations*, I am very desirous that you would give this a place in the next Number of your valuable Journal.

I remain, &c.

*December* 30, 1807. D. F. W.

## Figures and Tables

**Figure f1:**